# Family caregiver satisfaction with inpatient rehabilitation care

**DOI:** 10.1371/journal.pone.0213767

**Published:** 2019-03-15

**Authors:** Kristine T. Hanson, Kathleen F. Carlson, Greta Friedemann-Sanchez, Laura A. Meis, Courtney H. Van Houtven, Agnes C. Jensen, Sean M. Phelan, Joan M. Griffin

**Affiliations:** 1 Robert D. and Patricia E. Kern Center for the Science of Health Care Delivery, Mayo Clinic, Rochester, Minnesota, United States of America; 2 Center to Improve Veteran Involvement in Care, VA Portland Health Care System, Portland, Oregon, United States of America; 3 Oregon Health and Science University-Portland State University School of Public Health, Portland, Oregon, United States of America; 4 Hubert H. Humphrey School of Public Affairs, University of Minnesota, Minneapolis, Minnesota, United States of America; 5 University of Minnesota Medical School, Minneapolis, Minnesota, United States of America; 6 Center for Care Delivery and Outcomes Research, Minneapolis VA Healthcare System, Minneapolis, Minnesota, United States of America; 7 Durham VA Medical Center, Durham, North Carolina, United States of America; 8 Duke University School of Medicine, Durham, North Carolina, United States of America; 9 Division of Health Care Policy and Research, Mayo Clinic, Rochester, Minnesota, United States of America; University of Sussex, UNITED KINGDOM

## Abstract

**Introduction:**

Informal family caregivers play an increasingly important role in healthcare. Despite their role in ongoing management and coordination of care, caregiver satisfaction with the healthcare services care recipients receive has been understudied. We sought to assess what influences caregiver satisfaction with inpatient care provided to their care recipient among caregivers of veterans with traumatic brain injury (TBI) and polytrauma.

**Methods:**

Data from the Family and Caregiver Experience Survey, a national survey of caregivers of veterans with TBI and polytrauma, was used to explore factors associated with caregiver satisfaction with the care his/her care recipient received while an inpatient at a US Department of Veterans Affairs (VA) Polytrauma Rehabilitation Center. Caregiver and care recipient demographic and injury factors and potential addressable factors including social support, caregiver training received, and caregiver perceptions of being valued by the VA were evaluated for their associations with caregivers’ satisfaction with their care recipients’ healthcare.

**Results:**

The majority of the 524 caregivers reported being mostly or very satisfied with their care recipient’s inpatient care (75%, n = 393). Higher satisfaction with inpatient care was significantly associated with greater caregiver social support, receipt of training from the VA, and perceptions of being valued by the VA, both on univariate analysis and after controlling for care recipient TBI severity and caregiver’s relationship to the care recipient.

**Conclusions:**

Results suggest that supporting a strong social network for caregivers, providing caregiver training, and employing practices that communicate that family caregiving is valued by providers and healthcare organizations are promising avenues for improving caregiver satisfaction.

## Introduction

An estimated 43.5 million adults in the United States act as informal family caregivers [1], playing a vital role in healthcare. For people with cognitive deficits or who are heavily dependent on their caregivers for provision, coordination, and management of care, caregivers are often the conduit between the healthcare system and patients, acting either as a resource for or barrier to receiving timely care; therefore, involvement of family caregivers in decisions about the patient’s care and incorporation of family caregivers as part of the care team is especially important for achieving patient-centered care and ensuring continuity of patient care [2–5].

Amidst a rapidly evolving body of work on patient-centered care [6–10], there is a lack of literature assessing caregivers as key participants in that care. On the other hand, factors associated with caregiver health outcomes and quality of life are well-studied. There is strong evidence to suggest that improving social support and caregiver training (e.g., medication management, understanding the care recipient’s disease process) reduces caregiver burden, isolation, anxiety, and depression, and increases the quality of the caregiver’s life [11–16]. However, although a 2016 study of caregivers, patients, and their healthcare providers identified attentive, personalized, and family-centered care as key elements of care quality and satisfaction with care for patients and their families [17], factors that contribute to caregiver satisfaction with care recipients’ healthcare remain largely unknown. While satisfaction is likely both directly and indirectly determined by many factors, little is known about whether it varies by caregiver or care recipient demographics or by factors that may be addressable by health system interventions that focus on both patient and caregiver needs and values. Identification of factors that contribute to caregiver satisfaction with care could guide intervention efforts.

In this study we sought to examine caregiver satisfaction with inpatient healthcare services received by veterans who survived traumatic brain injury (TBI) and polytrauma during wars in the Middle East and then received inpatient rehabilitation care at one of five US Department of Veterans Affairs (VA) Polytrauma Rehabilitation Centers (PRC), which specialize in inpatient rehabilitation for multiple traumatic injuries [18–20]. Similar to caregivers of non-VA patients, a growing body of literature shows that caregivers of injured veterans act as both advocates and care managers for their care recipient, playing a critical role in veteran rehabilitation [3,21–25]. Assuming these roles, however, is not without costs. Research has shown that caring for someone with a TBI is stressful, especially for spouses, young families with children, and those with financial and medical needs, and that stress can be exacerbated by conflicts with medical teams [26,27]. McLaughlin, for example, found that greater conflict between staff and families led to less satisfaction among families of inpatient TBI program [28]. Similarly, Verhaeghe and colleagues (2005), in a review of the literature, also found that healthcare provider support can reduce stress and help families cope, but that conflict with healthcare team staff can induce stress, making it difficult to effectively cope and potentially impairing patient recovery [27].

Based on these findings and findings from previously published work showing that injury severity and demographic factors are associated with caregiver outcomes among caregivers of veterans with TBI [29], we hypothesized that caregiver and care recipient demographics and care recipients’ military service and injury details—factors that are individually fixed—are associated with caregivers’ satisfaction with inpatient care. Identifying sub-groups of caregivers with lower satisfaction may assist in targeting interventions. We also hypothesized that factors associated with the needs and values of the patient and their caregiver—including caregivers’ social support, receipt of needed training from the healthcare system, and caregiver endorsement of being valued by the healthcare system—influence higher caregiver satisfaction with inpatient care. These analyses were intended to identify potentially addressable, patient-centered care factors that could be targets of intervention to improve satisfaction.

## Methods

### Sample

The Family and Caregiver Experience Survey (FACES), a cross-sectional, mailed survey, was administered in 2009 to the next of kin of living military service members who met the following criteria: 1) served in the military during Operation Iraqi Freedom or Operation Enduring Freedom, 2) had a TBI and at least one other traumatic injury affecting another body system (e.g., fracture, hearing loss, vision loss, post-traumatic stress disorder), 3) received care at a PRC between September 2001 and February 2009 and were discharged to either an institution or community setting, and 4) had been discharged from a PRC for at least 3 months. Most were discharged to home following their inpatient stay, and, given the complexity and severity of injuries, many required ongoing support and care from family caregivers to function at a desired level [18]. Administration and methodology of the FACES survey has been previously described [18]. Original study protocols were reviewed and approved by Institutional Review Boards at all PRC sites. The conduct of this secondary analysis of de-identified data was reviewed and deemed exempt from Institutional Review Board committee review by the University of Minnesota and the Mayo Clinic.

### Measures

All self-reported survey items used in this analysis are displayed in S1 Table.

#### Outcome of interest

Inpatient caregiver satisfaction, our primary outcome, was assessed by asking, “Overall, how satisfied were you with the medical care your care recipient received while an inpatient at the VA Polytrauma Rehabilitation Unit?” Response options were “Very satisfied,” “Mostly satisfied,” “Somewhat dissatisfied,” “Mostly dissatisfied,” and “Extremely dissatisfied.” With the goal of providing actionable evidence for moving caregiver satisfaction from unfavorable to favorable, we defined a favorable satisfaction outcome as either “Very satisfied” or “Mostly satisfied*”* and an unfavorable satisfaction outcome as “Somewhat satisfied,” “Mostly dissatisfied,” or “Extremely dissatisfied.” For univariate analysis we combined “Mostly Dissatisfied” and “Extremely dissatisfied,” but we elected to leave “Somewhat satisfied” as a separate category in order to assess the characteristics of this group, as they may indicate a group more amenable to a favorable satisfaction response with intervention. For multivariable modeling, we further dichotomized caregiver satisfaction with inpatient care as favorable (Mostly/very satisfied) versus unfavorable (Somewhat satisfied/Mostly dissatisfied/Extremely dissatisfied) to facilitate the goal of moving all caregivers towards favorable satisfaction.

#### Fixed factors

Fixed factors included caregiver demographics (sex, race, ethnicity, highest level of education, relationship to the care recipient); care recipient demographics (sex, race, and ethnicity); and military service and injury details (injury location [Iraq/Afghanistan vs. US or elsewhere], time since injury, and severity of the initial TBI).

Response options for relationship to the care recipient included husband, wife, girlfriend/boyfriend (romantic partner), son, daughter, mother, father, sister, brother, friend, and other. Responses were categorized for analysis as parent, spouse/romantic partner, and all other.

Severity of the initial TBI was assessed by the caregiver’s report of the patient’s length of loss of consciousness at the time of initial TBI/polytrauma and categorized as mild TBI (≤ 30 minutes), moderate TBI (>30 minutes to <7 days), and severe TBI (≥ 7 days) [30]. The variable categories were then recoded as mild versus moderate/severe TBI.

#### Potentially addressable factors

Social support, caregiver training, and caregiver perceptions of being valued by the healthcare system were identified as factors that could be targets for intervention (amended or modified) if associated with caregiver satisfaction.

Caregiver social support was measured by a modified version of the ENRICHD social support instrument, a validated measure of social support used to assess the availability of emotional and instrumental support [31] with good internal reliability (Cronbach’s α = 0.86). Higher ENRICHD sum scores indicate greater social support.

To assess caregiver training, caregivers were asked if they received training from the VA in five domains: navigating the VA or Department of Defense benefits or medical system; administering medication or helping with medication side effects; helping with the care recipient’s pain; supporting the care recipient’s emotions or feelings; and helping with the care recipient’s assistive devices (i.e., palm pilots and other vision, hearing, or memory aids). Possible responses for each training question were “yes,” “no,” or “not needed.” For caregivers who answered all five training domains, the training questions were also combined into a single summary variable which had good internal reliability (Cronbach’s α = 0.82). The variable had the following values: “at least one needed training provided,” “no needed training provided,” and “no training needed” (all five training domains were not needed).

Three questions developed by the study team about the perception that VA values caregiver well-being were asked, with responses ranging from 1 (disagree a lot) to 5 (agree a lot): (1) VA cares about the caregiver’s well-being, (2) VA recognizes the importance of the caregiver’s role as caregiver, and (3) VA trusts how the caregiver cares for their care recipient. We conducted an exploratory factor analysis and, with orthogonal rotation, found the three items loaded onto one factor (Eigenvalue = 2.54), suggesting the 3 items represented a common construct that we labeled “valued by the VA.” Therefore, we created a single scale from the three items and used the summed score (range 3–15) to assess the degree to which the caregiver felt valued by the VA. Higher scores reflected feeling more valued by the VA. Scores were coded by quartiles (3–8, 9–10, 11–14, and 15) to represent levels of lowest to highest feelings of being valued. The internal reliability of this scale was good (Cronbach’s α = 0.91).

### Statistical analysis

Associations between independent variables and satisfaction with inpatient care were assessed using chi-square tests or Fisher’s exact tests for categorical variables and Kruskal-Wallis tests for continuous variables. Multivariable logistic regression was used to assess the relationship between potentially addressable factors and caregiver satisfaction with inpatient care after controlling for caregiver and care recipient demographics. One model that included social support, receipt of at least one domain of caregiver training, and caregiver perceptions of being valued by the VA was used to assess the association of these addressable factors with inpatient care after adjusting for relationship of caregiver to care recipient and TBI severity. Further models explored the five training domains as separate variables. Caregiver and care recipient characteristics that were associated with caregiver satisfaction at p<0.10 in univariate analysis were selected for inclusion in the multivariable models. These models were intended to be hypothesis-generating, rather than causal (etiologic) or predictive, in order to inform future research. Results from logistic regression are reported using odds ratios (OR) and 95% confidence intervals (CI).

All statistical tests were two-sided, and p-values less than 0.05 were considered statistically significant. All statistical analyses were performed using SAS version 9.4 (SAS Institute Inc., Cary, NC).

## Results

Out of 1045 identified caregivers, a total of 564 caregivers responded to the survey; of these, 524 reported their satisfaction with their care recipient’s inpatient care and comprised the study sample. The majority (80%) of caregivers were female. Most were parents of care recipients (N = 319, 61%), while 173 (33%) were spouses or romantic partners, and 32 (6%) were siblings, grandparents, other relatives, friends, or other relations. Time since injury was less than 4 years in 43% of care recipients. Further details about the full cohort of caregiver and care recipients are described by Griffin and colleagues [18].

### Satisfaction with inpatient care

The majority of caregivers (75%, n = 393) reported being mostly or very satisfied with their care recipient’s inpatient care at the VA PRC (27%, n = 144 mostly satisfied and 48%, n = 249 very satisfied), while 13% (n = 66) were somewhat satisfied and 12% (n = 65) were mostly or extremely dissatisfied (8%, n = 40 mostly dissatisfied and 5%, n = 25 extremely dissatisfied).

#### Caregiver and veteran factors

Shown in Table 1, caregivers who were the parent of or other relation to the care recipient were more likely to be mostly or very satisfied with care compared to spouses/partners to the care recipient. Similarly, the association between TBI severity and satisfaction was approaching significance (p = 0.08), suggesting that caregivers of veterans with moderate or severe injury severity may be more satisfied with care recipient inpatient care compared to caregivers of veterans with mild injury severity.

**Table 1:** Fixed factors versus caregiver satisfaction with inpatient care.

	Overall N (column %)	Caregiver Satisfaction with Inpatient Care N (row %)			
		Mostly/ Extremely Dissatisfied	Somewhat Satisfied	Mostly/Very Satisfied	p-value
**N (%)**	524 (100)	65 (12.4)	66 (12.6)	393 (75.0)	
**Caregiver Demographics**					
**Caregiver’s relationship to care recipient**					0.03^*b*^
Parent	319 (60.9)	35 (11.0)	32 (10.0)	252 (79.0)	
Spouse/Romantic partner	173 (33.0)	28 (16.2)	30 (17.3)	115 (66.5)	
Other (sibling, grandparent, other relative, friend)	32 (6.1)	6 (18.8)^*a*^		26 (81.3)	
**Sex**					0.71^*b*^
Male	103 (20.0)	10 (9.7)	14 (13.6)	79 (76.7)	
Female	411 (80.0)	52 (12.7)	52 (12.7)	307 (74.7)	
**Race**					0.11^*b*^
White only indicated	384 (80.7)	52 (13.5)	46 (12.0)	286 (74.5)	
Non-white or more than 1 race	92 (19.3)	7 (7.6)	17 (18.5)	68 (73.9)	
**Ethnicity**					0.18^*b*^
Non-Latino/Hispanic	437 (88.8)	52 (11.9)	63 (14.4)	322 (73.7)	
Latino/Hispanic	55 (11.2)	11 (20.0) ^*a*^		44 (80.0)	
**Marital status**					0.22^*b*^
Married/living with partner	396 (77.2)	54 (13.6)	51 (12.9)	291 (73.5)	
Divorced/separated/widowed/ never married	117 (22.8)	9 (7.7)	15 (12.8)	93 (79.5)	
Missing	11	2	0	9	
**Highest year of education**					0.25^*b*^
Less than high school graduate or HS graduate	130 (25.8)	14 (10.8)	24 (18.5)	92 (70.8)	
Some college or trade school	236 (46.8)	27 (11.4)	28 (11.9)	181 (76.7)	
Bachelor’s degree or higher	138 (27.4)	20 (14.5)	14 (10.1)	104 (75.4)	
**Care Recipient Demographics**					
**Sex**					0.79^*c*^
Male	499 (95.2)	61 (12.2)	63 (12.6)	375 (75.2)	
Female	25 (4.8)	7 (28.0) ^*a*^		18 (72.0)	
**Race**					0.36^*b*^
White	276 (85.7)	39 (14.1)	37 (13.4)	200 (72.5)	
Non-white or more than 1 race	46 (14.3)	10 (21.7) ^*a*^		36 (78.3)	
**Ethnicity**					1.00^*c*^
Non-Latino/Hispanic	324 (92.0)	40 (12.3)	43 (13.3)	241 (74.4)	
Latino/Hispanic	28 (8.0)	7 (25.0) ^*a*^		21 (75.0)	
**Care Recipient Service and Injury Details**					
**Injury location**					0.31^*b*^
Iraq or Afghanistan	250 (47.7)	32 (12.8)	37 (14.8)	181 (72.4)	
United States or location other than Iraq/Afghanistan	274 (52.3)	33 (12.0)	29 (10.6)	212 (77.4)	
**Time since injury**					0.11^*b*^
1–3 years	218 (43.0)	19 (8.7)	26 (11.9)	173 (79.4)	
4–6 years	226 (44.6)	32 (14.2)	26 (11.5)	168 (74.3)	
7 years or more	63 (12.4)	8 (12.7)	13 (20.6)	42 (66.7)	
**Severity of TBI**					0.08^*b*^
Mild	154 (29.6)	22 (14.3)	26 (16.9)	106 (68.8)	
Moderate/Severe	367 (70.4)	43 (11.7)	39 (10.6)	285 (77.7)	

^*a*^ Low cell sizes have been collapsed for display for privacy purposes, but data were analyzed with 3-level satisfaction for all variables

^*b*^ Chi-square test

^*c*^ Fisher’s exact test

#### Potentially addressable factors

Greater social support was associated with increased satisfaction with inpatient care. ENRICHD sum scores among those who were mostly or very satisfied (median 28, IQR 21–32) with inpatient care were significantly higher than scores among caregivers who were somewhat satisfied (median 21, IQR 16–26) or mostly or extremely dissatisfied (median 22, IQR 17–29) (p<0.001; Table 2).

**Table 2:** Addressable factors versus caregiver satisfaction with inpatient care.

	Overall N (column %)	Caregiver Satisfaction with Inpatient Care N (row %)			
		Mostly/ Extremely Dissatisfied	Somewhat Satisfied	Mostly/Very Satisfied	p-value
**N (%)**	524	65 (12.4)	66 (12.6)	393 (75.0)	
**Social support**					<0.001^*b*^
Median (Interquartile range)	26 (19,32)	22 (17,29)	21.2 (16,26)	28 (21,32)	
Range	7 to 35	11 to 35	7 to 35	7 to 35	
**Received training in navigating the VA or Department of Defense benefits or medical system**					0.001^*c*^
No	252 (51.4)	45 (17.9)	37 (14.7)	170 (67.5)	
Yes	164 (33.5)	10 (6.1)	19 (11.6)	135 (82.3)	
Not needed	74 (15.1)	5 (6.8)	8 (10.8)	61 (82.4)	
**Received training in administering medication or help with medication side effects**					0.001^*c*^
No	172 (35.0)	34 (19.8)	28 (16.3)	110 (64.0)	
Yes	183 (37.3)	17 (9.3)	20 (10.9)	146 (79.8)	
Not needed	136 (27.7)	10 (7.4)	15 (11.0)	111 (81.6)	
**Received training in helping with care recipient's pain**					<0.001^*c*^
No	164 (33.7)	35 (21.3)	27 (16.5)	102 (62.2)	
Yes	168 (34.6)	13 (7.7)	20 (11.9)	135 (80.4)	
Not needed	154 (31.7)	11 (7.1)	16 (10.4)	127 (82.5)	
**Received training in supporting care recipient's emotions or feelings**					<0.001^*c*^
No	206 (42.0)	42 (20.4)	35 (17.0)	129 (62.6)	
Yes	220 (44.9)	14 (6.4)	18 (8.2)	188 (85.5)	
Not needed	64 (13.1)	5 (7.8)	10 (15.6)	49 (76.6)	
**Received training in helping with care recipient's assistive devices (eg, vision, hearing, language or memory aids)**					0.01^*c*^
No	155 (32.1)	29 (18.7)	26 (16.8)	100 (64.5)	
Yes	159 (32.9)	16 (10.1)	15 (9.4)	128 (80.5)	
Not needed	169 (35.0)	16 (9.5)	21 (12.4)	132 (78.1)	
**Valued by VA**					<0.001^*c*^
3–8 (lowest feelings of being valued)	131 (27.0)	33 (25.2)	32 (24.4)	66 (50.4)	
9–10	119 (24.5)	12 (10.1)	11 (9.2)	96 (80.7)	
11–14	119 (24.5)	10 (8.4)	14 (11.8)	95 (79.8)	
15 (highest feelings of being valued)	116 (23.9)	10 (8.6)^*a*^		106 (91.4)	

^*a*^ Low cell sizes have been collapsed for display for privacy purposes, but data were analyzed with 3-level satisfaction for all variables

^*b*^ Kruskal-Wallis test

^*c*^ Chi-square test

Satisfaction with inpatient care varied by receipt of caregiver training. While just under half (48.6%) of caregivers reported either receiving or not needing training in navigating the VA or Department of Defense benefits or medical system, over half of caregivers received or did not need the other four types of training that were assessed: administering medicine or helping with medication side effects (65.0%), helping with care recipient’s pain (66.3%), supporting their care recipient’s emotions or feelings (58.0%), and helping with their care recipient's assistive devices like vision, hearing, language or memory aids (67.9%). Caregivers who received training or did not need training were significantly more satisfied with inpatient care than those who did not receive training for all five types of training (all p<0.05; Table 2).

Caregivers who expressed a higher perception of being valued by the VA were significantly more satisfied with their care recipient’s inpatient care, where 91.4% of caregivers with a score of 15 were mostly or very satisfied with care recipient care, compared to 79.8% of caregivers with a score of 11–14, 80.7% of caregivers with a score of 9–10, and 50.4% of caregivers with a score of 3–8 (p<0.001; Table 2).

#### Multivariable analysis

After adjusting for caregiver kinship to care recipient and TBI severity, greater social support (OR 1.19 for an increase of 5 units in the ENRICHD sum score, 95% CI 1.00–1.40), receiving needed training in at least one domain (OR 2.02 vs no needed training provided, 95% CI 1.21–3.37), and stronger perceptions of being valued by the VA (OR 5.86 for highest perception vs lowest perception of being valued, 95% CI 2.65–13.00) were significantly associated with higher odds of caregiver satisfaction with inpatient care (Table 3). In a second model with the same factors and each of the five training domains included as separate variables (in contrast with the composite described above), the only training domain that was significantly, uniquely associated with higher odds of caregiver satisfaction with inpatient care was training in supporting the care recipient’s emotions or feelings (OR 2.17, 95% CI 1.08–4.37). Stronger perceptions of being valued by the VA (OR 5.15 for highest vs lowest perception of being valued, 95% CI 2.23–11.88) remained significantly associated with satisfaction with inpatient care, but social support was no longer significantly associated with satisfaction with inpatient care (S1 Table).

**Table 3:** Associations between addressable factors and satisfaction with inpatient care in a multivariable logistic regression model ^a^.

		Mostly/very satisfied with inpatient care	
		OR (95% CI)	p-value
Social support	Per increase of 5 units	1.19 (1.00–1.40)	0.046
Any training provided by the VA	At least one needed training provided	2.02 (1.21–3.37)	0.008
	No needed training provided	(Ref)	
	No training needed	1.21 (0.41–3.57)	0.73
Valued by VA	3–8	(Ref)	
	9–10	3.27 (1.74–6.14)	<0.001
	11–14	2.84 (1.49–5.41)	0.002
	15	5.86 (2.65–13.00)	<0.001

^***a***^ This model included all three addressable factors displayed in the table and adjusted for kinship to care recipient and veteran TBI severity

## Discussion

In this study assessing caregiver satisfaction among veterans treated as inpatients in a VA PRC, the majority of caregivers (75%) were satisfied with their care recipient’s inpatient care. Caregiver dissatisfaction was associated both with fixed factors, including relationship to the care recipient, and with potentially addressable factors, including lack of social support, lack of caregiver training received, and low caregiver perceptions of being valued by the healthcare system. These associations suggest opportunities for healthcare organizations, like the VA, and healthcare teams to improve caregiver satisfaction.

This study’s findings suggest that dissatisfaction with care is associated with unmet caregiver needs. Caregivers with low levels of social support at the time of the survey reported lower satisfaction with care. Social support has been shown to be an important caregiver need that influences caregiver well-being [16,32] where greater social support was associated with lower burden, isolation, and disappointment among caregivers of patients with TBI one year after injury [14]. Therefore, improving a caregiver’s support network, perhaps through support groups, presents an opportunity for the healthcare system to respond to caregiver needs for social support. This is especially important with regards to spouse caregivers since spousal caregivers were found to be more likely to have lower satisfaction with inpatient care. Spousal caregivers of people with TBI in other studies and in previous reports of this study sample have been shown to have higher rates of stress [27,28], and it is possible that stress is also associated with lower satisfaction. Caregivers of people with TBI, therefore, may especially benefit from targeted interventions to improve their satisfaction with care.

Lower caregiver perceptions of the VA healthcare system valuing the caregiver role were also found to be associated with lower caregiver satisfaction with care, an association that remained after controlling for social support. Caregiver feelings of being valued may be influenced by the degree to which caregivers felt they were active and valued participants in their veterans’ care, a concept key to patient-centered care. Improving caregivers’ sense of value with the healthcare team, however, may not always be straight-forward. Dubbed by McLaughlin as an ‘adversarial alliance,’ healthcare teams are often responsible for building support with families and teaching them about patients’ goals of care and TBI rehabilitation efforts, yet they must also candidly communicate the often discouraging realities about prognosis [28]. Testing and implementing strategies to include caregivers as valued members of a patient’s care team and reduce the inherent adversity may provide the VA and other healthcare systems an opportunity to improve caregiver satisfaction with care recipient care. Future research should examine the role of the quality and quantity of caregiver participation in the care team in explaining caregiver satisfaction.

Receipt of training was also associated with caregiver satisfaction. Previous studies have shown that caregiver training, be it psychotherapeutic, psychoeducational interventions, or skill development training, may improve caregiver skills and knowledge about the care recipient’s condition and also reduce caregiver burden, depression, and anxiety, and improve caregiver quality of life [11,15,33–35]. In this study, even after controlling for injury severity, caregiver relationship to care recipient, social support, and perceptions of feeling valued by the healthcare system, caregivers who received needed training in at least one domain were twice as likely to be satisfied compared to those who did not receive any needed training. Additionally, when all training was included in multivariable analyses, only training to address the care recipient’s emotional needs was independently associated with satisfaction, making that a particularly important target for optimizing caregiver satisfaction among patients with TBI. Implementation of practices and processes that assess caregiver training needs and then provide that training could communicate to caregivers that they are valued by the healthcare system as part of the care team and improve caregiver satisfaction with care.

Some of these factors are indeed now being addressed within the VA healthcare system. In 2010, after the FACES survey was administered, Public Law 111–163, the Caregivers and Veterans Omnibus Health Services Act, was passed. This law gave the VA expanded authority to support relatives who care for seriously injured post-9/11 veterans [36]. The Program of Comprehensive Assistance for Family Caregivers offers a monthly stipend and other types of assistance such as training, counseling, expanded access to mental healthcare and respite care, and access to caregiver support coordinators at all VA medical centers [37,38]. As of September 2015, over 27,000 family caregivers were enrolled in the program [38] indicating that there is high demand for caregiver services. While enrollment in the program has been associated with improved access to primary care and mental healthcare for veterans [38], it is unknown whether the program is associated with improvements in caregiver satisfaction with care. However, based on the results of our study, it stands to reason that caregivers who are enrolled in the program may have higher satisfaction with care than those who are not. Our study provides baseline evidence upon which follow-up assessments of caregiver satisfaction may be compared. The current study, and any future studies, may also support the development of similar programs in healthcare systems outside of the VA.

In spite of our unique and potentially generalizable findings, there are some limitations to this work. First, this study used only one question for inpatient satisfaction instead of a longer, validated scale. Therefore, we did not capture what factors of the inpatient experience caregivers considered when assessing care recipient medical care. Although there is a no consensus on how best to measure experiences of healthcare delivery [39], it is possible that factors commonly studied in patient experience studies like communication with providers, responsiveness of nursing staff, quality of facilities, and care transition experiences were important. Furthermore, our measure of caregiver satisfaction with inpatient care could reflect a complex combination of caregiver and care recipient factors, including unmet needs of either the caregiver or care recipient, unrealistic caregiver expectations about their care recipient’s course of clinical rehabilitation or social integration, or caregiver burden. Further research is warranted to explore not only what factors caregivers take into account when assessing their satisfaction with care recipient care, but also factors that contribute to that satisfaction. Using qualitative research methods, these factors may be elucidated to understand how caregivers experience and define inpatient care [39]. While studies have assessed factors associated with caregiver satisfaction derived from the caregiving experience [40,41], there is a paucity of evidence addressing factors that contribute to caregiver satisfaction with the formal care received by their care recipients. A strength of this study was our ability to examine both fixed and addressable factors and their association with caregiver satisfaction; our results may serve as a foundation upon which future research may build.

Another potential limitation to this study is its reliance on cross-sectional data, limiting our assessment of cause and effect as well as our ability to track changes in caregiver satisfaction over time. Recall bias may have affected our estimated measures of association, as the caregiver’s current satisfaction with outpatient care, among other intervening events in the time that passed between the care recipient’s inpatient stay and the survey, may have affected how the caregiver perceived their satisfaction with inpatient care. However, almost half of caregivers responded to the survey within 1–3 years of their care recipient’s injury, helping mitigate this potential limitation. Furthermore, the association between time since injury and caregiver satisfaction with care was not significant. Ongoing assessments of satisfaction and needs among informal caregivers of traumatically injured patients would help identify changes over time and, potentially, drivers of these changes.

Finally, it is also possible that non-responders to our survey were more or less likely to be satisfied with their care. Our previous research with this sample shows that the care recipients of non-responding caregivers were significantly more likely to have lower functional status at admission and discharge from the PRC [42].

Despite these limitations, our study provides an important new look into factors associated with caregiver satisfaction with inpatient care received by the care recipient. While preliminary in nature, results of our work can inform future research that explores caregiver satisfaction in greater granularity, at the point of care receipt as well as longitudinally over time.

## Conclusions

In an era in which family caregivers play an increasingly vital role in healthcare while patient-centered care is in focus on the national healthcare stage, it is of utmost importance to understand the experience and needs of the caregiver, particularly among patients with cognitive deficits and those dependent on family caregivers. This study suggests that providers looking to improve caregiver satisfaction with care should focus on interventions that support a strong social network for caregivers, provide caregiver training, and ensure that caregivers feel valued by healthcare providers and institutions.

## Supporting information

S1 Table Survey items from the family and caregiver experience survey (FACES) included in this analysis. Excerpt of items in the full FACES survey.
(DOCX)

S2 Table Associations between addressable factors and satisfaction with inpatient care in a multivariable logistic regression model, considering each training domain as a separate variable. Model included the addressable factors displayed in the table and adjusted for kinship to care recipient and veteran TBI severity.
(DOCX)
