# Supplementary material for: Family caregiver satisfaction with inpatient rehabilitation care
Source: PLoS One. 2019 Mar 15;14(3):e0213767. doi: 10.1371/journal.pone.0213767 (PMC6420024; doi:10.1371/journal.pone.0213767)
Supplement: S1 Table — Excerpt of items in the full FACES survey. (DOCX) [file pone.0213767.s001.docx]

**S1 Table. Survey items from the Family and Caregiver Experience Survey (FACES) included in this analysis.** Excerpt of items in the full FACES survey.

| **ABOUT YOU** | | | | | | | | | | | |
| --- | --- | --- | --- | --- | --- | --- | --- | --- | --- | --- | --- |
| Are you male or female? | - Male - Female | | | | | | | | | | |
| Which of the following best describes you? *Choose all that apply.* | - American Indian or Alaska Native - Asian - Black or African American - Native Hawaiian - Other Pacific Islander - White - Other   Please specify: ___________________ | | | | | | | | | | |
| Are you of Latino/Hispanic origin? | - Yes - No - Don’t Know/Unsure | | | | | | | | | | |
| Are you currently married, separated, divorced, widowed or never married? *Choose only one.* | - Married - Divorced - Living with a partner - Separated - Widowed - Never married | | | | | | | | | | |
| What is the highest grade of school you have completed? *Choose only one.* | - Less than high school - Some high school - Graduated from high school/received GED - Vocational, business or trade school - Some college - Associates degree (AA/AS) - Bachelor’s degree (BA/BS) - Master’s or Doctoral Degree (MA/MS, MD, PhD) or other post-graduate training | | | | | | | | | | |
| **ABOUT YOUR CARE RECIPIENT ^1^** | | | | | | | | | | | |
| Where did your care recipient get injured? | - In the US - In Iraq - In Afghanistan - Overseas, but not in Iraq or Afghanistan   In which country? ___________________ | | | | | | | | | | |
| When was your care recipient injured? | __________________ __________________  Month Year | | | | | | | | | | |
| Due to your care recipient’s injury, was he/she unconscious/in a coma for any length of time (not including medically induced coma)? If yes, for how long? | - Was not unconscious/in a coma - Was unconscious/in a coma for 30 minutes or less - Was unconscious/in a coma for more than 30 minutes but less than 1 week - Was unconscious/in a coma for 1 week or more - Don’t know/unsure | | | | | | | | | | |
| Overall, how satisfied were you with the medical care your care recipient received while an inpatient at the VA Physical Rehabilitation Unit? | - Very satisfied - Mostly satisfied - Somewhat satisfied - Mostly dissatisfied - Extremely dissatisfied | | | | | | | | | | |
| **THE CARE YOU PROVIDE** | | | | | | | | | | | |
| How are you related to the person you are helping (your “care recipient”)? | - I am her husband. - I am his wife. - I am his/her girlfriend/boyfriend (romantic partner). - I am his/her son. - I am his/her daughter. - I am his/her mother. - I am his/her father. - I am his/her sister. - I am his/her brother. - I am his/her friend. - Other relationship, not listed above.   Please specify: ___________________ | | | | | | | | | | |
| Did any VA health care provider give you any training or show you how to…   - navigate the VA or Department of Defense benefits or medical system? - administer medication or help with medication side effects? - help with your care recipient’s pain? - support your care recipient’s emotions or feelings? - help with your care recipient’s assistive devices, such as palm pilots or other vision, hearing, language or memory aids? | YES  □  □  □  □  □ | | | NO  □  □  □  □  □ | | | | | NOT NEEDED  □  □  □  □  □ | | |
| **YOU AND YOUR RELATIONSHIPS** | | | | | | | | | | | |
| How much do you agree or disagree with the following statements?  The VA…   - cares about my care recipient’s well being. - cares about my well being. - recognizes the importance of my role as caregiver. - trusts how I care for my care recipient. | DISAGREE  A LOT  □  □  □  □ | | DISAGREE  A LITTLE  □  □  □  □ | | | NEITHER AGREE NOR DISAGREE  □  □  □  □ | | AGREE A LITTLE  □  □  □  □ | | | AGREE A LOT  □  □  □  □ |
| The next question contains statements about how often you feel you have supported in your role as a caregiver. Please answer based on how things are currently.  In the past 7 days…   - Was there someone available to whom you can count on to listen to you when you need to talk? - Was there someone available to give you good advice about a problem? - Was there someone available to you who shows you love and affection? - Was there someone available to help with daily chores? - Could you count on anyone to provide you with emotional support (talking over problems or helping you make a difficult decision)? - Did you have as much contact as you would like with someone you feel close to, someone in whom you can trust and confide in? | NONE OF THE TIME  □  □  □  □  □  □ | A LITTLE OF THE TIME  □  □  □  □  □  □ | | | SOME OF THE TIME  □  □  □  □  □  □ | | MOST OF THE TIME  □  □  □  □  □  □ | | | ALL OF THE TIME  □  □  □  □  □  □ | |

^1^ Care recipient demographics (….) were abstracted from the medical record. Location of injury and length of loss of consciousness survey response data were supplemented with data from the medical record.
