# Supplementary material for: Family caregiver satisfaction with inpatient rehabilitation care
Source: PLoS One. 2019 Mar 15;14(3):e0213767. doi: 10.1371/journal.pone.0213767 (PMC6420024; doi:10.1371/journal.pone.0213767)
Supplement: S2 Table — Model included the addressable factors displayed in the table and adjusted for kinship to care recipient and veteran TBI severity. (DOCX) [file pone.0213767.s002.docx]

**S2 Table. Associations between addressable factors and satisfaction with inpatient care in a multivariable logistic regression model, considering each training domain as a separate variable.**

|  |  | **Mostly/very satisfied with inpatient care** | |
| --- | --- | --- | --- |
|  |  | **OR (95% CI)** | **p-value** |
| Social support: ENRICHD sum score | Per increase of 5 units | 1.15 (0.97-1.36) | 0.12 |
| Training: navigate the VA or Department of Defense benefits or medical system | Yes | 1.13 (0.58-2.18) | 0.73 |
|  | No | (Ref) |  |
|  | Not needed | 1.79 (0.61-5.21) | 0.29 |
| Training: administer medication or help with medication side effects | Yes | 1.05 (0.51-2.17) | 0.89 |
|  | No | (Ref) |  |
|  | Not needed | 0.69 (0.28-1.72) | 0.43 |
| Training: help with your care recipient's pain | Yes | 1.06 (0.50-2.23) | 0.88 |
|  | No | (Ref) |  |
|  | Not needed | 2.29 (1.01-5.20) | 0.048 |
| Training: support your care recipient's emotions or feelings | Yes | 2.17 (1.08-4.37) | 0.03 |
|  | No | (Ref) |  |
|  | Not needed | 0.62 (0.23-1.62) | 0.33 |
| Training: help with your care recipient's assistive devices | Yes | 1.07 (0.51-2.24) | 0.85 |
|  | No | (Ref) |  |
|  | Not needed | 0.99 (0.50-1.94) | 0.97 |
| Valued by VA | 3-8 | (Ref) |  |
|  | 9-10 | 3.12 (1.62-5.98) | <0.001 |
|  | 11-14 | 2.51 (1.28-4.89) | 0.007 |
|  | 15 | 5.15 (2.23-11.88) | <0.001 |

Model included the addressable factors displayed in the table and adjusted for kinship to care recipient and veteran TBI severity
